# Automated measurement of the spontaneous tail coiling of zebrafish embryos as a sensitive behavior endpoint using a workflow in KNIME

**DOI:** 10.1016/j.mex.2021.101330

**Published:** 2021-04-04

**Authors:** Afolarin O. Ogungbemi, Elisabet Teixido, Riccardo Massei, Stefan Scholz, Eberhard Küster

**Affiliations:** a Department of Bioanalytical Ecotoxicology, Helmholtz Centre for Environmental Research-UFZ, Permoserstraße 15, Leipzig 04318, Germany; b Institute for Environmental Sciences, University of Koblenz-Landau, 76829, Fortstraße 7, Landau, Germany; c Department of Effect-Directed Analysis, Helmholtz Centre for Environmental Research - UFZ, Permoserstraße 15, Leipzig 04318, Germany

**Keywords:** Developmental neurotoxicity, Behavior toxicology, Spontaneous activity, Hyperactivity, Hypoactivity, Alternatives to animal testing

## Abstract

Neuroactive substances are the largest group of chemicals detected in European surface waters. Mixtures of neuroactive substances occurring at low concentrations can induce adverse neurological effects in humans and organisms in the environment. Therefore, there is a need to develop new screening tools to detect these chemicals. Measurement of behavior or motor effects in rodents and fish are usually performed to assess potential neurotoxicity for risk assessment. However, due to pain and stress inflicted on these animals, the scientific community is advocating for new alternative methods based on the 3R principle (reduce, replace and refine). As a result, the behavior measurement of early stages of zebrafish embryos such as locomotor response, photomotor response and spontaneous tail coiling are considered as a valid alternative to adult animal testing. In this study, we developed a workflow to investigate the spontaneous tail coiling (STC) of zebrafish embryos and to accurately measure the STC effect in the KNIME software. We validated the STC protocol with 3 substances (abamectin, chlorpyrifos-oxon and pyracostrobin) which have different mechanisms of action. The KNIME workflow combined with easy and cost-effective method of video acquisition makes this STC protocol a valuable method for neurotoxicity testing.

• Video acquisition duration of 60 s at 25 ± 1 hpf was used
• 20 embryos exposed per dish and acclimatized for 30 min before video acquisition
• Capability to inspect and correct errors for high accuracy

## Graphical abstract

**Figure:**
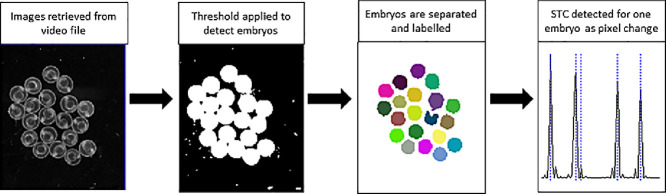

Specifications Table

**Table:** 

Subject Area:	Agricultural and Biological Sciences
More specific subject area:	Ecotoxicology; Neurotoxicology; Behavior toxicology
Method name:	Spontaneous tail coiling test with zebrafish embryos
Name and reference of original method:	Saint‐Amant, L. and Drapeau, P., 1998. Time course of the development of motor behaviors in the zebrafish embryo. Journal of neurobiology, 37(4), pp.622–632. https://doi.org/10.1002/(SICI)1097–4695(199,812)37:4%3C622::AID-NEU10%3E3.0.CO;2-S
Resource availability:	KNIME workflow: https://hub.knime.com/elisabet_t/spaces/Public/latest/Spontaneous%20tail%20coilings-measurement KNIME setup*:*https://youtu.be/wgJN71zTvRw

## Background

The spontaneous tail coiling (STC) represents the earliest motor activity observed in the developing neural network of zebrafish embryos. It is assumed to be mediated by the innervation of the muscle by the primary motor neurons, which are first present at around 17 hpf [5,11,13]. These motor neurons are known to originate in the spinal cord. In contrast, another early motor behavior, the photomotor response requires a high-intensity light stimulus which is mediated in the hindbrain [10]. A comparison between the PMR test and the STC test has been previously published [6]. Counting the average STC in the embryos is considered to be a fast and reliable behavioral endpoint which finds its application in the screening of neuroactive substances or for general toxicological screening [7]. A previous review has also identified the STC test to be more sensitive to detect organophosphate insecticides in comparison to other commonly used behavior tests such as locomotor activity and photomotor response [6]. Different software are available to measure the STC, however, most of them are expensive and may not allow for a manual correction or quality check of the assessment (i.e. identification of embryos not labeled appropriately). As a result, the actual count of the STC may be inadequately assessed. The aim of the current method paper is to describe a way to accurately and automatically count the STC using a workflow in the open and free KNIME software.

## Embryo selection and exposure

**Fig. 1:**
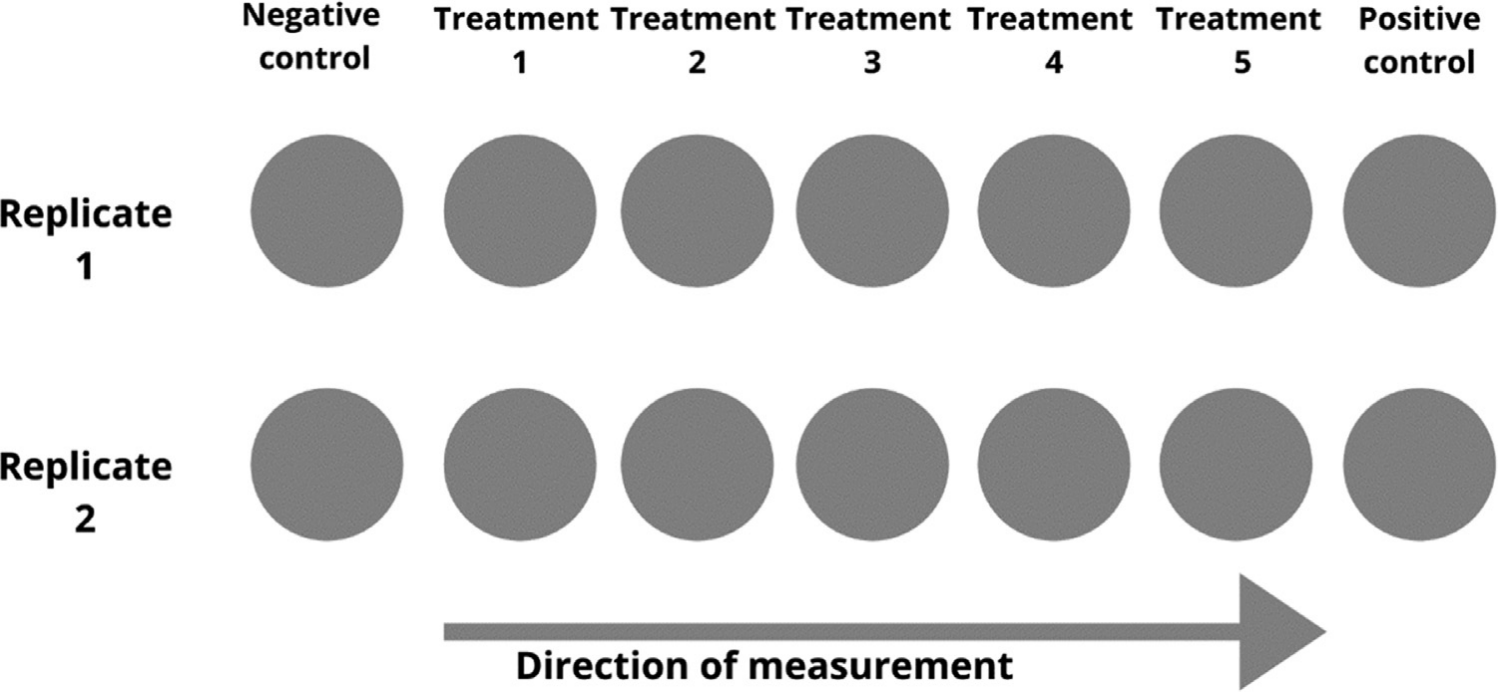
Visual representation of the arrangement of the exposure glass dishes and the direction of measurement during video acquisition. The measurement starts with all treatments of replicate 1 followed by replicate 2.

Adult zebrafish (OBI and WIK strains) were obtained from a local commercial breeder and crossed to obtain a hybrid strain (OBI-WIK, F3 generation). Fish were cultured under 14 h light/10 h dark photoperiod in 120 L aquaria. Spawning trays were placed in the tanks on the afternoon 4–6 h before the end of the light cycle. The following day, lights were automatically switched on at 8am to initiate the spawning and eggs were collected at 9am. In order to remove dirt and debris, the eggs were washed several times with ISO water. After washing, fertilized eggs between 2 and 3 hpf were selected under a stereomicroscope. Twenty embryos were exposed to 20 mL of test chemical in 40 mL glass petri-dish (60 mm diameter, Carl Roth GmbH, Karlsruhe Germany) and covered with a glass lid to prevent evaporation or cross-contamination. Glass dishes were incubated at 28 °C till the next day. Exposure was conducted with 14 glass dishes representing 7 concentrations and 2 replicates including the negative and positive control (Fig. 1). The glass dish exposure system allowed high resolution video-taping and was therefore preferred over multi-well plates. However, provided that a sufficient resolution can be provided for video recording of the entire well or individual wells, the protocol could be adopted for multi-well plates.

Specific procedure:

(1) ISO water was prepared according to ISO 7346–3 (1996) [80 mM CaCl_2_·2H_2_O, 20 mM MgSO_4_·7H_2_O, 31 mM NaHCO_3_, 3.1 mM KCl]
(2) Chemical stock solutions were prepared a day before in ISO water or were already standing on the bench when prepared in dimethyl sulfoxide (DMSO). The stock solution was diluted to give lower concentrations in 50 mL standard volumetric flasks.
(3) The glass petri-dishes were labeled with the necessary experiment information according to the number of treatments and replicates
(4) Twenty fertilized embryos (2–3 hpf) were selected and transferred into each labeled dish. Embryos were transferred into the dishes systematically i.e. first replicate for all treatments starting from lowest to highest concentration were filled and followed by the second replicate. This system models the video acquisition format (see Fig. 1). This ensures that similar time offset is transferred from the embryo selection to the video acquisition phase.
(5) The selected embryos were exposed to each chemical concentration and ISO water as negative control. Water was first removed using a pipette and 20 mL of the respective exposure solution was added into the dishes. A solvent control should be used when solvents are used to prepare the chemical solution.
(6) The dishes were covered and Incubated at 28 °C overnight.

## Video acquisition

On the next day, the microscope and camera were setup for video acquisition. The embryos were removed from the incubator and allowed to acclimatize at room temperature for at least 30 min. The acclimatization period aimed to ensure a constant measuring temperature since the microscope was not contained within an incubator. We found in a previous study [7] that this equilibration temperature of 30 min enabled the reproducible assessment of STC. Beginning at 9AM ± 0.25 h, each glass dish was videotaped for 1 min. The videotaping was done systematically such that the first replicate of each concentration starting from negative controls to highest concentration was recorded, followed by the second replicate (Fig 1). Additionally, the negative controls could be recorded again at the end to ensure the stability of the STC measurement.

**Fig. 2:**
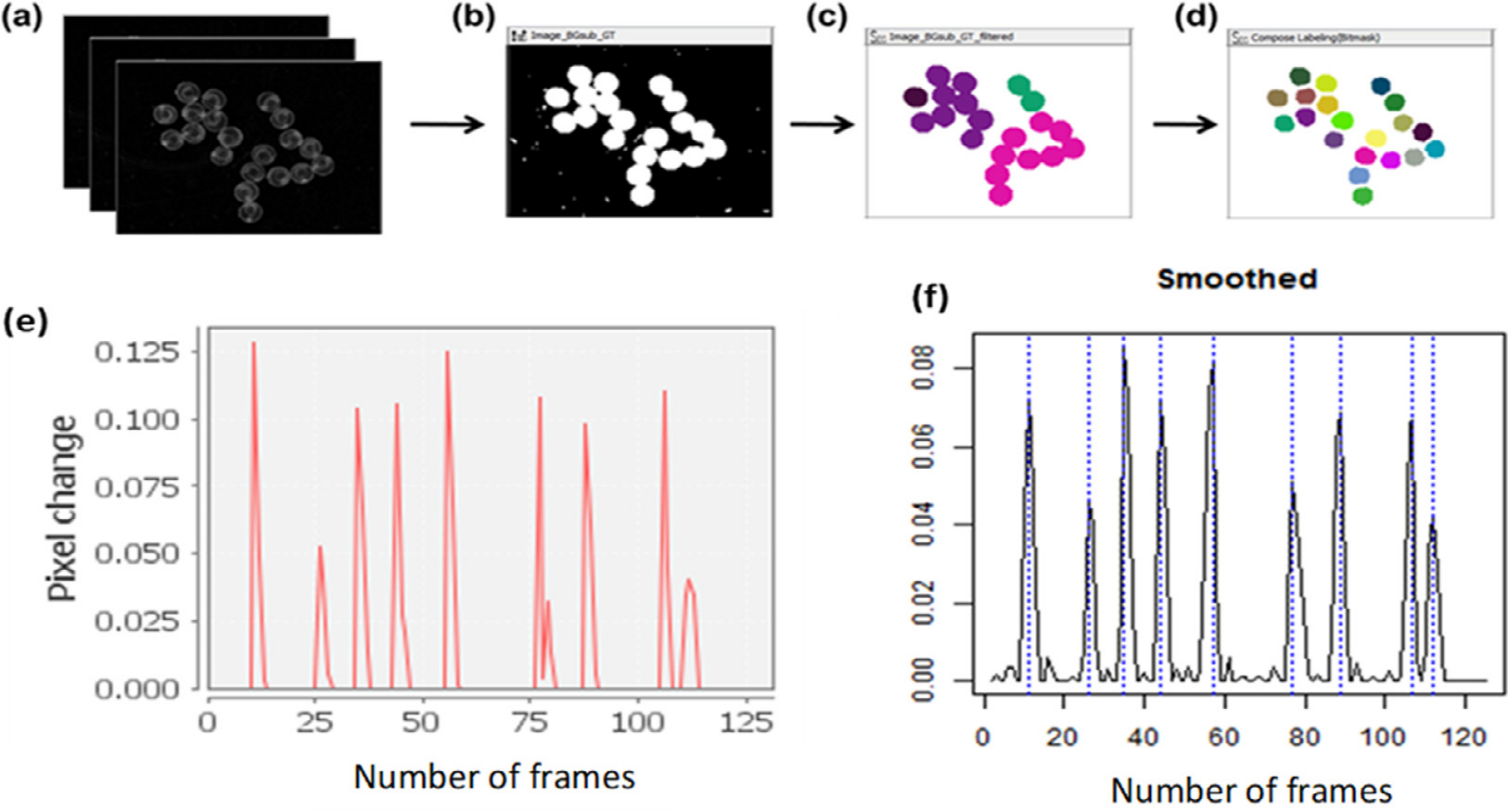
Automated workflow for STC analysis in KNIME. (a) Video files (AVI format) are converted to image stacks. (b) A threshold is applied to identify the location of the embryos in the image, (c) the binary image is segmented and (d) adjacent touching embryos are separated using morphological operations. (e) Variance of pixels between frames are identified to indicate movement. The graph shows the variance of one selected embryo. (f) Each peak (indicated by a dashed vertical line) represents an individual tail flip. Peaks were identified using an R script embedded in a KNIME workflow. The plot show the STC peaks of one embryo within a duration of 60 s or 120 frames.
(7) Embryos were videotaped for 1 min using a stop clock. Embryos could be monitored or observed during video acquisition via the computer screen. It is important to keep the table holding the camera still during video recording.
(8) Video acquisition was completed within a period of ≈ 30 min in order to avoid confounding effects of developmental stage within an experiment i.e. 2 min per dish or 28 min for 7 treatments and 2 replicates.
(9) A second chemical exposure may be conducted in parallel by following the same procedure as for the first chemical i.e. preparation of chemical solutions, embryo selection may be conducted simultaneously but video acquisition should be performed in blocks of independent experiments.
(10) The videos were stored in a mobile drive and transferred to a local or cloud drive for further analysis.

Specific procedure:

(1) The computer screen, camera (Olympus DP21) and stereomicroscope (Olympus SZX7) were turned-on. Light source was from a LED illumination base of the microscope.
(2) The magnification of the microscope was set to 0.8X and the background base was tuned to dark background to create a contrast against the transparent embryos.
(3) Camera settings were ISO = 400; shutter speed = 1/80, image size = 400 × 300 pixels and image resolution = 1600 × 1200 pixels. Other types of camera including a mobile phone camera may be used to collect videos if quality requirements are met.
(4) Embryos at ≈ 24–25 h post fertilization were removed from the incubator and equilibrated at room temperature for at least 30 min.
(5) Embryos were assessed for developmental malformations and lethality under the stereomicroscope. Deformed or dead embryos were removed or separated. Number of removed embryos were recorded in a data sheet.
(6) All normal embryos were clustered to the center by slightly swirling the dish and forceps were used to improve the clustering when required. Embryos were placed side by side and not super imposed on each other (Fig. 2a).

## KNIME setup and analysis

### Description of the KNIME® workflow

**Fig. 3:**
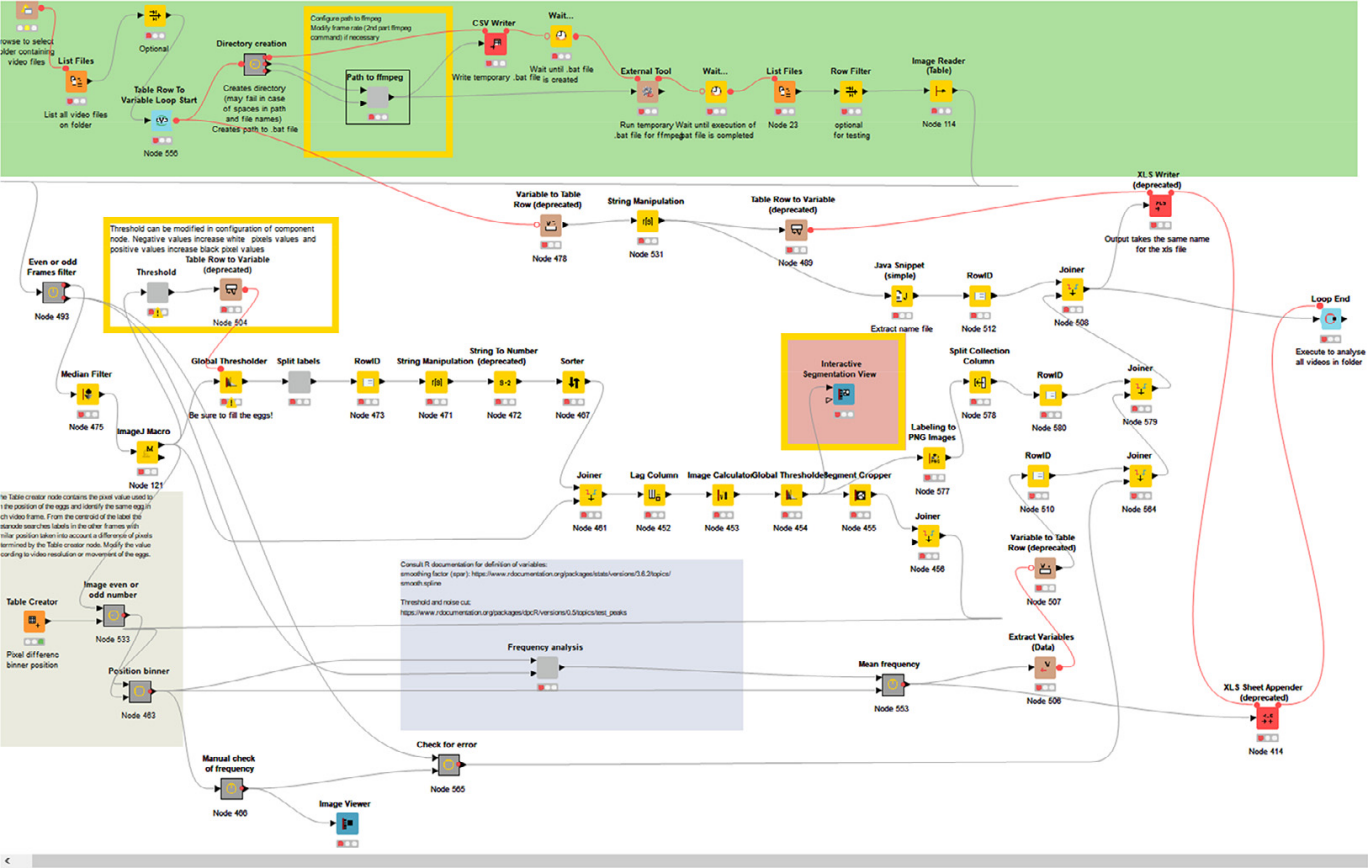
The STC workflow in KNIME.

The workflow (Fig 3) computes how many times an embryo moves by image analysis using the video recordings. The workflow is divided in several sections using “metanodes” representing a collection of several other nodes, each responsible for a specific calculation (basic description of the KNIME® Analytical Platform can be consulted in [1,3]).

The workflow requires video recordings in AVI format as input and iterates over all video files in the selected folder. The video files are converted to stacks of images using the FFmpeg tool (2016) at a sample rate of 120 frames per minute. The program is executed by using the external tool node in KNIME®. The individual embryos in the image are detected by applying a threshold for conversion to binary images. Briefly, for each frame a median filter is applied to smooth the image and the background is subtracted using the ImageJ macro node (SubstractBackground function). Then a threshold is automatically set and applied using a variable node. The threshold is based on the mean pixel intensity of all frames, but it can be adjusted depending on the characteristics of the video files. The image with the applied threshold should display embryos completely filled in white and the background in black (Fig. 2b). Then images are segmented to label each embryo independently. In order to separate connected labels of adjacent embryos, the Waehlby cell clump splitter node in KNIME® is used [16]. Subsequently, various morphological image operations (e.g. erosion) are applied to optimize segmentation of individual embryos (Fig. 2c–d).

Subsequently the variance of gray values of embryo labels of two successive images is compared to identify movements. Therefore, a lag column is created and the difference of pixel across each video frame stamp is calculated using the image calculator node. Then a threshold is applied and the variance in pixel is extracted using the image segment features node for each labeled embryo. This threshold was set by verifying the concordance between the final KNIME® output and the visual count of the STCs.

Embryo labels between subsequent frames were associated using the centroid of the label. Because embryos may slightly move between each frame, a distance of 15 pixels was allowed between label centroids of individual frames.

Finally the frequency of movements of each embryo is analyzed by iterating over each label and using as input the pixel variance over all the frames. An increase in variance indicates tail coiling. Therefore during the time series, peaks representing tail coilings were identified using the function ‘findpeaks’ of the R package ‘quantmod’ [12] embedded in the KNIME workflow by means of an R snippet node. Fig. 2e–f shows an example of a graph obtained and the identified peaks for each embryo. The STC frequency per minute is calculated taking into account the total duration of the video recordings and at the end, an Excel® file with the same name as the video is automatically saved with the output results.

Note that we included a metanode (“Check for error”) after the “position binner” metanode. This node serves as an internal control to detect when there is an error during the image analysis, for example in case that embryo were not correctly identified and labeled. That would require to repeat the analysis of the specific video by adjusting the first threshold value (the one that allows identifying each individual embryo) or correcting the maximum distance of centroid allowed between individual frames.

### Specific procedure

More details on how to implement the workflow are shown in the youtube video: https://youtu.be/wgJN71zTvRw

(1) Download KNIME (https://www.knime.com/downloads) and install on your PC.
(2) Also download the STC workflow and ffmpeg software from KNIME hub and import it into your KNIME software. Open the KNIME software and set the workspace to your preferred folder.
(3) Install all necessary KNIME extensions (math formula, quickforms, community image analysis nodes, external tool node) for the STC workflow and restart KNIME.
(4) Configure ffmpeg software by setting up the path to its location on your PC using the bin folder as the end of the path.
(5) Open R software and install the required packages (quantmod, dpcR and Rserve). If necessary, change the file path of R to the correct location. R error messages can be diagnosed by clicking Eval script in KNIME and then installing the missing packages
(6) The video file or folder containing the video files to analyze should be inserted in the KNIME workspace folder, use the Explorer browser node to select the folder containing the video. Alternatively, you can use the List files node to select folders outside of the workspace folder. Specific video files from the folder can be selected using the row filter node next to list files node.
(7) The workflow can be executed by clicking the double green arrow in the top menu to start the loop.
(8) The workflow creates a folder with the same name as the video file analyzed which contains the stack of images generated. After the analysis they should be removed to save disk space.
(9) Changing KNIME parameters:
  (a) Frames per minute can be changed in the ffmpeg component configuration and in the Frequency analysis component configuration. Default is set to 120 frames per minute. Both configurations should have the same frame rate for a correct analysis.
  (b) R parameters for threshold and smoothing can be changed in the Frequency analysis component configuration. Default is set to 0.003 and 0.1 for threshold and smoothing respectively.
  (c) Global threshold for detecting embryo can be changed by subtracting or adding a number in the Threshold component configuration. This controls the global threshold node.

## Data treatment

Data was obtained from the KNIME workflow as the number of STCs per minute or STC frequency for one embryo. Fig 2f shows an example of the peak count of an embryo with 9 STC counts per min. The workflow automatically calculates the STC frequency per min based on the length of the video. However, it is also possible to manually calculate the STC frequency especially when a correction is required. A correction protocol may be implemented when the user makes observations that may potentially influence the outcome of the STC analysis i.e. uncontrolled events such as strong signal from movement of whole embryo, many weak peaks close to the peak defining threshold and inaccurate accountability of fast multiple peaks may influence the STC frequency.

### Correction protocol

**Fig. 4:**
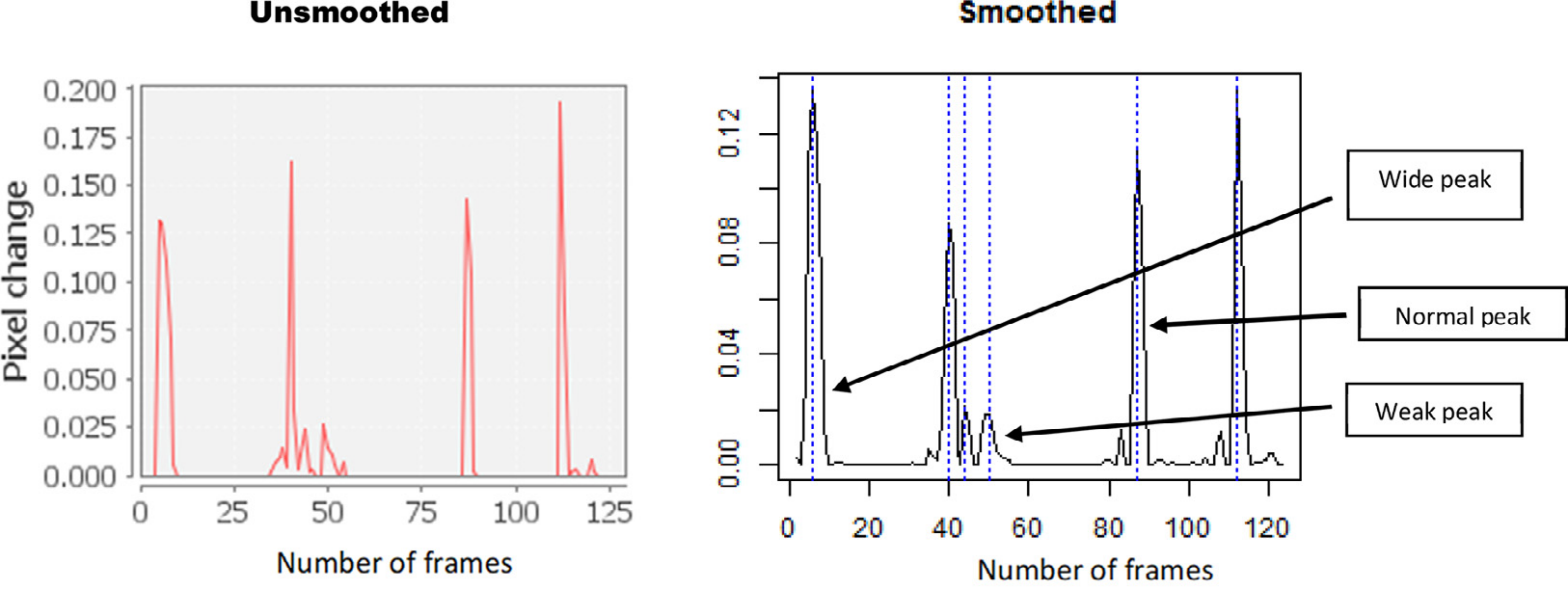
Smoothed and unsmoothed STC peaks. Unsmoothed peaks show the raw peaks without any processing and can be used to validate errors. The wide peaks could be due to fast multiple coils while the weak peaks could be due to movement of whole embryo.

Inspect the peaks: A possible correction protocol could be to visually inspect the peaks for errors e.g. by comparing unsmoothed and smoothed peaks (Fig 4). Irregular shaped, wide peaks and very small peaks are suspects. For example, the suspected wide and weak peaks shown in Fig 4 can be confirmed by inspecting the unsmoothed peaks which display the shape of the erroneous peaks more clearly.

**Fig. 5:**
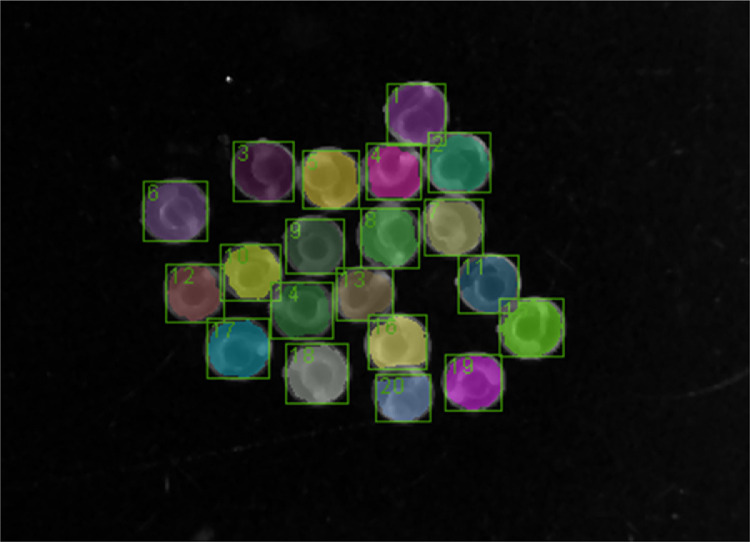
Labeling and annotation of individual embryos. Labels can be used to identify embryos in the video to check for suspected erroneous peaks. Note: Labels in the excel sheet start from 0 while labeled images start from 1. Therefore, embryo 0 in the excel sheet will be embryo 1 in the labeled image.

**Fig. 6:**
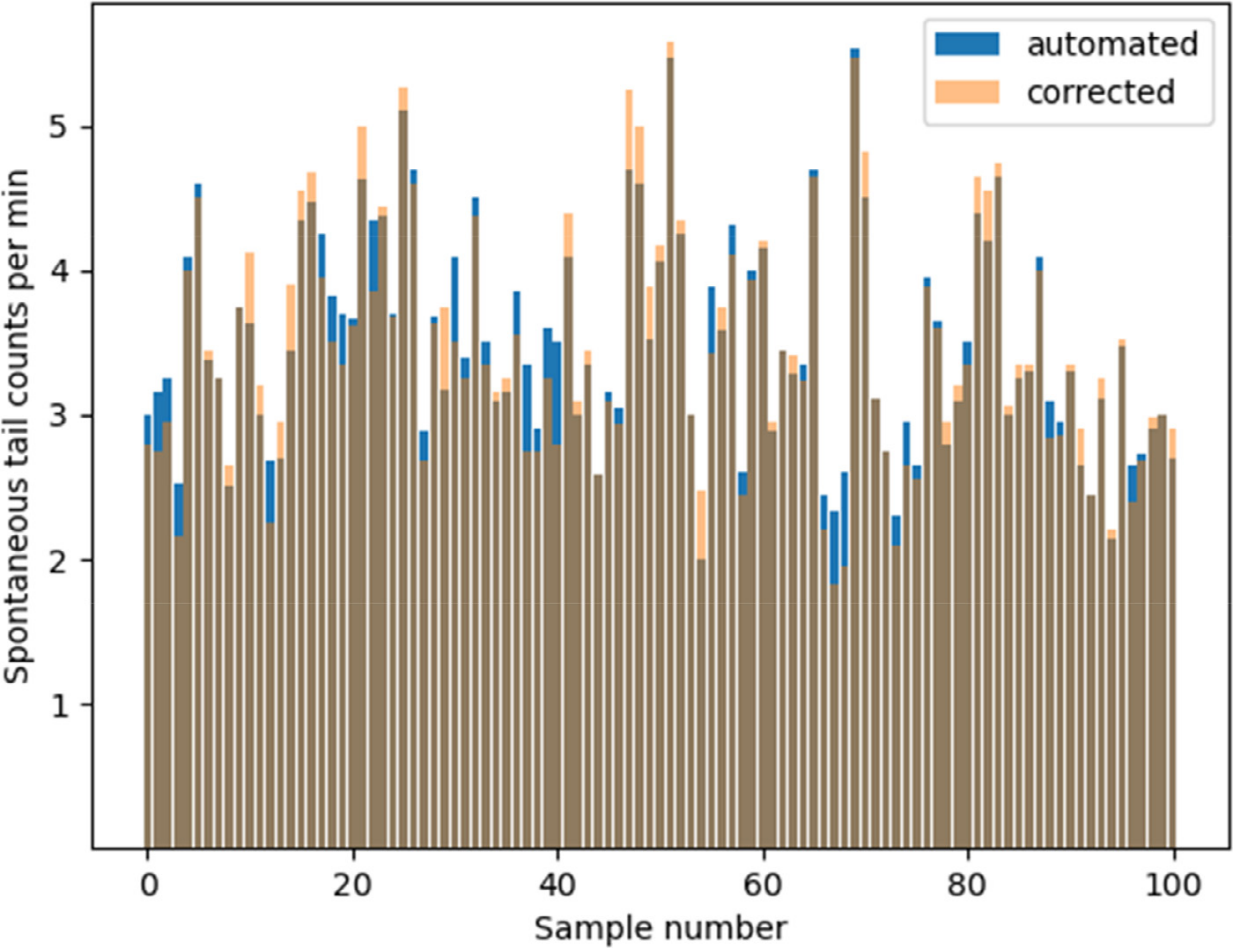
Comparison of automated and corrected STC counts for 100 independent control measurements. Each bar or peak represents the mean STC count for 20 embryos. Dark brown portion of the bars represent areas where automated and corrected counts are the same. Blue portion of the bar represents tests in which automated counts are higher than corrected while yellow portion represents tests in which corrected are higher. (For interpretation of the references to color in this figure legend, the reader is referred to the web version of this article.)

Suspect peaks: Suspected peaks should be verified using the annotated label of the embryo to locate and check embryo movement in the original video (Fig 5). The user would be able to identify errors without checking the original video after a period of video training to identify error peaks. A visual comparison of automated and corrected STC counts for control measurements in different independent experiments shows that the differences are not significant (Fig 6). Therefore, the results from the automated analysis may be used for fast screening of chemicals and correction may only be required for a thorough analysis such as mode of action identification analysis.

### Data analysis

The STC frequency of the individual embryos, the mean of all embryos, the STC peaks and the annotated embryos are compiled as results within an output Excel file from the KNIME workflow. An example Excel file and corresponding video is given in the supplementary information (SI Excel file 1 and video 1). The mean STC frequency (3.3 ± 0.85/min) for untreated embryos varied between independent experiments (Fig 6). To obtain comparable STC results for independent experiments of the same chemical, we had to normalize the STC frequency for different concentrations to that of the untreated embryos to obtain a normalized percentage mean STC frequency.

To determine the actual effect of a chemical, it is possible to analyze the data using concentration-response modeling or hypothesis testing. We performed concentration-response modeling to estimate the EC_50_ – the concentration at which the percentage STC is half-maximum relative to the untreated embryos. Hypothesis testing may also be used when sufficient technical replicates are tested. Shapiro test and Bartlett test could be used to check for normality and homogeneity of variance, respectively. In case normality of the data is not met, non-parametric test such Kruskal-Wallis or Dunnet tests could be used to test for statistical differences between treatment groups. Statistical difference was considered when the *p*-value < 0.05.

## Method validation

The STC test as devised in this study can be used to screen neuroactive chemicals based on the hyper and hypoactivity response of zebrafish embryos. In addition, the STC test may also be used to screen non-neuroactive substances assuming that behavior endpoints are usually more sensitive than lethality. The STC test method described in the current paper have already been applied to screen a range of 18 test chemicals with different modes of action [7]. Here we give 3 examples of chemicals with typical modes of action either with an expected hyperactivity or hypoactivity or without any expected effect.

Abamectin is an avermectin insecticide expected to cause hypoactivity by activating Gamma aminobutyric acid-gated chloride channel. Abamectin induced hypoactivity in the STC test at an EC_50_ of 0.055 µM. Four other studies reported hypoactivity for abamectin in the STC test but the reported lowest observed effect concentrations (LOEC) were higher than the EC_50_ found in the current study [8,9,15,17]. This could be due to conducting exposure in plastic well-plates rather than glass as exposure vessel. The only study [8] that conducted exposure in glass had the least deviation (factor of 4) from our study. Abamectin is highly lipophilic (logDpH7.4(ACD/Labs) of 5.85) and hence has more affinity to bind to plastic than glass, therefore, abamectin may be more bioavailable to the embryos in a glass container leading to effects occurring at lower concentration. The use of a different endpoint (percentage of organisms showing hypoactivity) could be an additional reason for the deviations in effect concentrations [6]. The only study [17] that used the same endpoint (STC frequency) as in the present study had the second least deviation of a factor 6. Further, the analysis duration used in these studies were lower than the 1 min used in the present study and this could also be the cause for inconsistent effect concentrations.

Chlorpyrifos-oxon is a metabolite of chlopyrifos which is an organophosphate insecticide. It acts by inhibiting acetylcholinesterase enzyme which breaks down acetylcholine, and therefore keeps the nicotinic acetylcholine receptors open for sodium ions to flow into the cell leading to an action potential and hence potential hyperactivity. In our study, chlorpyrifos-oxon induced hyperactivity in the STC test at an EC_50_ and EC_10_ of 0.32 and 0.05 µM respectively and this is consistent (the EC_10_) with the LOEC of 0.03 µM reported by Weichert et al. [17]. These effect concentrations are significantly lower (or more toxic) than that of the parent compound – chlorpyrifos which might be due to the limited bioactivation in the early stages of zebrafish embryo [7].

**Fig. 7:**
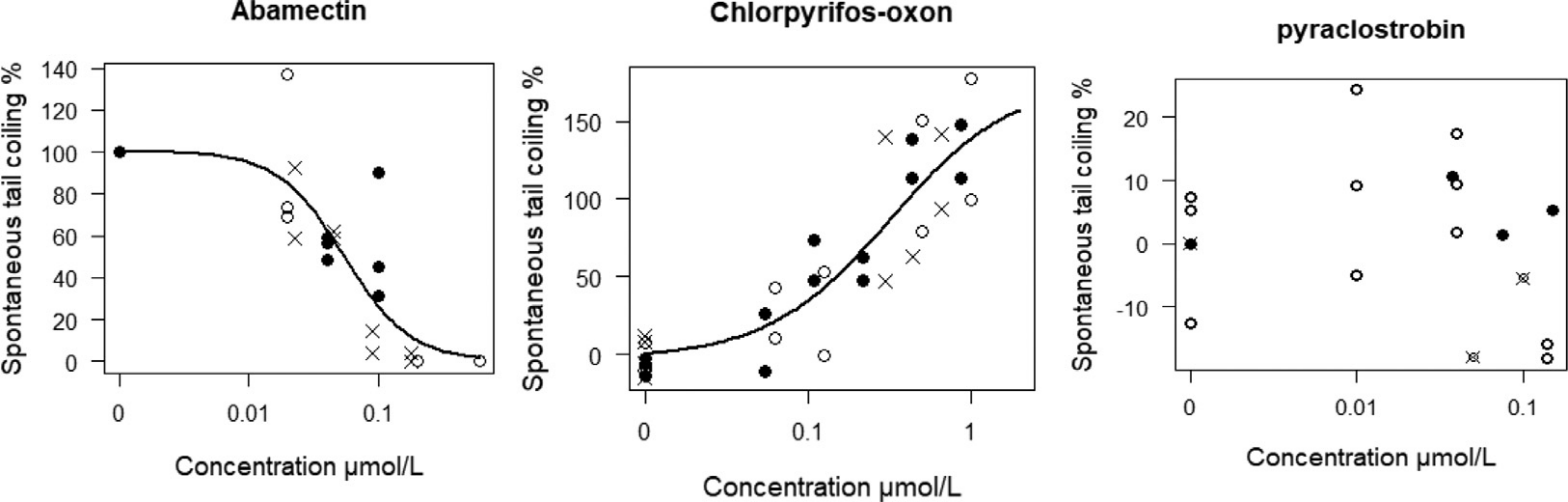
Concentration-response curves for abamectin, chlorpyrifos and pyraclostrobin. Y-axis represents spontaneous tail coiling normalized to control and X-axis shows the exposure concentration. Different symbols represent independent experiments. Upward curves indicate hyperactivity effect with respect to controls while downward curves indicate hypoactivity effect. Figures taken from [7].

Pyraclostrobin is a fungicide and expected to not impact on the STC due to its classification as a narcotic or baseline toxic in quantitative structure and activity relationship (QSAR) for zebrafish [2]. Pyraclostrobin did not induce any effect in the STC test up to a concentration of 0.15 µM and similar absence of STC effect up to 0.76 µM were reported by [9]. Fig. 7 shows the concentration-response relationships for abamectin, chlorpyrifos-oxon and pyraclostrobin. These results validate the STC test method for screening chemicals.

## Conclusion

In this paper, we described a protocol for measuring spontaneous tail coiling in zebrafish embryos. First, we gave exhaustive guidelines on how to conduct the experiment based on optimized experimental parameters. Second, we detailed how to automatically analyze the collected video recordings in a workflow with the open source KNIME software. Third, we then validated the described method using three chemicals with different modes of action. The STC test can now be used for the assessment of neuro (developmental) toxicity and testing of both neuro and non-neuroactive compounds. This automated analysis in KNIME provides an easier way to analyze STC over the laborious manual counting in [17]. The correction protocol utilized in the current study could enable more accurate estimation of the STC counts than other advanced behavioral tools that may not allow for real-time inspection and correction of STC peaks (eg. [9]). However, costume tools which are free and solely targeted at STC analysis are recently being developed. In this regard, the KNIME workflow developed in the current study alongside a MATLAB® tool [4] and an Image J macro [18] are the current available freeware for STC analysis to our knowledge. Due to video resolution of multi-well system, our STC assessment was developed for a microscope set-up which might reduce throughput. However, we are recently applying improved video resolutions and modified workflows to enable also the assessment of STC in multi-well plates (i.e. [14]). It is also important to recognize that the STC workflow presented in this article does not require an expensive imaging device and can be easily implemented with a camera mounted on a standard dissection microscope. This provides a cost-effective solution for laboratories that would like to add a new, simple and sensitive method to their repertoire of testing tools.

Supplementary material and/or Additional information:

SI Excel file 1 – Result output of the STC analysis in KNIME. Sheet 1 (“default”) contains embryo identification labels, total embryos analyzed and threshold of analysis. Sheet 2 (“Raw data”) contains the STC peaks, peak number for each embryo and STC frequency.

SI Video 1 – Video recording of 20 embryos which was analyzed in KNIME to give the result output in SI Excel file 1.

## Direct Submission or Co-Submission

Co-submissions are papers that have been submitted alongside an original research paper accepted for publication by another Elsevier journal

Co-Submission

NTT_106918

## Declaration of Competing Interest

The authors declare that they have no known competing financial interests or personal relationships that could have appeared to influence the work reported in this paper.

## Appendix Supplementary materials

Image, application 1

Image, video 2

Image
