# Supplementary figures and images for: Automated measurement of the spontaneous tail coiling of zebrafish embryos as a sensitive behavior endpoint using a workflow in KNIME
Source: MethodsX. 2021 Apr 4;8:101330. doi: 10.1016/j.mex.2021.101330 (PMC8374338; doi:10.1016/j.mex.2021.101330)

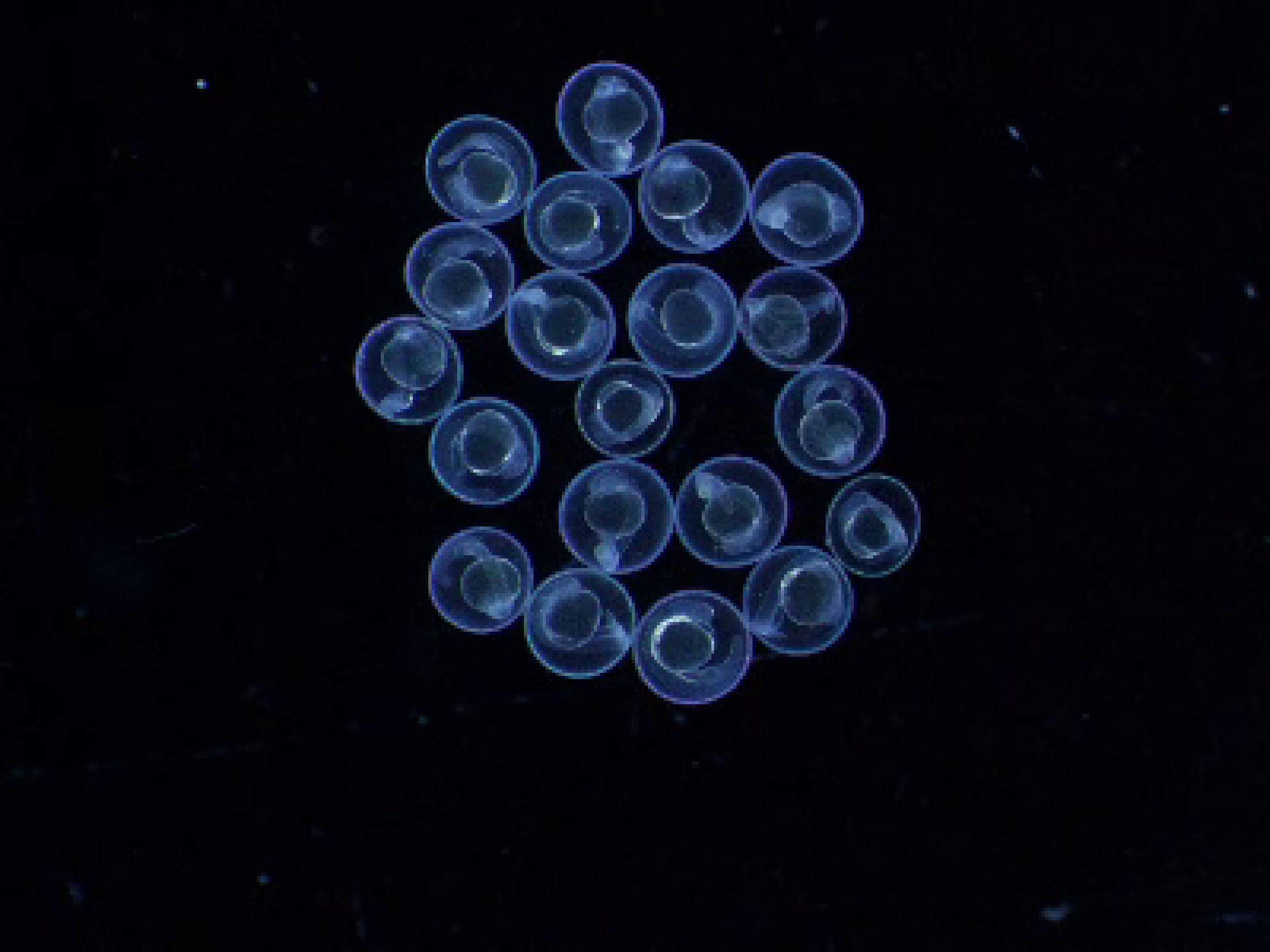

Supplement: Supplementary file 3 [file mmc3.jpg]
